# Supplementary material for: Accentuating the positive and eliminating the negative: Efficacy of TiO2 as digestibility index marker for poultry nutrition studies
Source: PLoS One. 2023 Jun 26;18(6):e0284724. doi: 10.1371/journal.pone.0284724 (PMC10292697; doi:10.1371/journal.pone.0284724)
Supplement: S1 Table — (DOCX) [file pone.0284724.s001.docx]

**Supporting Information**

**S1 Table. Ingredient composition and calculated nutrient concentrations of the basal diet.**

| Ingredient | Starter | Nutrient | Calculated |
| --- | --- | --- | --- |
| Wheat | 63.12% | Crude protein (%) | 21.55 |
| Soybean meal^1^ | 30.59% | Poultry AME kcal/kg | 2961.14 |
| Soy oil | 2.70% | Calcium (%) | 0.95 |
| Salt | 0.35% | Total phosphate (%) | 0.73 |
| DL Methionine | 0.17% | Available phosphate^3^ (%) | 0.45 |
| Lysine HCl | 0.12% | Phytate P (%) | 0.23 |
| Limestone | 0.95% | Crude fat (%) | 4.11 |
| Dicalcium Phosphate | 1.50% | Poultry ME MJ/kg | 12.39 |
| Vitamin premix^2^ | 0.50% | Poultry NE Kcal/kg | 1952.36 |

^1^48% minimum declared crude protein; sourced from USA.

^2^Vitamin and Mineral Premix content (per kg diet): Manganese 100 mg, zinc 88 mg, iron 20 mg, copper 10 mg, iodine 1 mg, magnesium 0.48 mg, selenium 0.2 mg, retinol 13.5 mg, cholecalciferol 3 mg, tocopherol 25 mg, menadione 5.0 mg, thiamine 3 mg, riboflavin 10.0 mg, pantothenic acid 15 mg, pyroxidine 3.0 mg, niacin 60 mg, cobalamin 30 µg, folic acid 1.5 mg, biotin 125 µg.

^3^ Available phosphate (%) does not account for phytate P contribution
