# Supplementary material for: Accentuating the positive and eliminating the negative: Efficacy of TiO2 as digestibility index marker for poultry nutrition studies
Source: PLoS One. 2023 Jun 26;18(6):e0284724. doi: 10.1371/journal.pone.0284724 (PMC10292697; doi:10.1371/journal.pone.0284724)
Supplement: Attachment — Submitted filename: Comment for editor - PLOS ONE (1).docx [file pone.0284724.s003.docx]

**Comment for the author: Major Review**

The manuscript “Accentuating the positive and eliminating the negative: "Efficacy of TiO_2_ as a digestibility index marker for poultry nutrition studies" presented by Sprigg et al, required a review. Manuscript included important information to support the proposal that the use of TiO_2_ as inert in the phosphorus digestibility trials, especially with enzyme evaluations. Are necessary corrections and clarifications to paper be accepted. The points to be corrected/clarified are detailed below:

Abstract: highlight important points of the material and methods (e.g., experimental period and age of the birds).

Introduction: Reduce the explanation about the different types of markers. Explain how phytase acts on liberation of inositol, and the interaction between titanium and inositol in the cell in pH acid. I recommended improve the objective sentence at the end of this section.

Line 208-223: State the age of the birds.

Line: 212-213: Writing this sentence “In brief, 480 male Ross 308 hatchlings were allocated randomly to 48 floor pens, with 6 replicate pens of 10 birds per treatment (8 treatment groups total)”. Give to the reader a sentence easier to understand how many birds per replication.

Line 213-214: The experimental design and treatment definition is very confusing and needs a better description. The authors mentioned that the experiment was conducted under a factorial arrangement (2 x 4), that’s meaning that has two factors of variability with two levels for the first factor and four levels for the second factor. This information no makes sense with “2 TiO_2_ level, control, and inositol plus and 3 phytase level”, since was used only one level of TiO2 (line 218), and 2 levels of phytase (line 223), etc. Additionally, the factors and treatments described in the M&M section are not the same that is those described in the result section (Table 1).

Line 211, 215, and 218: All of these lines have basal diet information that could be grouped together and summarized in a single sentence.

Line 231-232: Please, detail the procedure used for samples laboratory conduction (standard AOAC procedure? time, and temperature for freeze drying?).

Line 108-112: This section is being performed as a discussion, and required to improve with other studies or literature information to affirm that TiO_2_ has chemical proprieties to interact with phosphate components changing on the pH of the tract-gizzard.

Discussion:

Discuss in order to explain the use of titanium as inert in function of your observed results to show the importance of this marker, mainly when is phytase is used.
