# Supplementary material for: Accentuating the positive and eliminating the negative: Efficacy of TiO2 as digestibility index marker for poultry nutrition studies
Source: PLoS One. 2023 Jun 26;18(6):e0284724. doi: 10.1371/journal.pone.0284724 (PMC10292697; doi:10.1371/journal.pone.0284724)
Supplement: Attachment — Submitted filename: Response to Reviewers.docx [file pone.0284724.s004.docx]

21 December 2022

Dear Aziz ur Rahman Muhammad

Re. Accentuating the positive and eliminating the negative: efficacy of TiO_2_ as digestibility index marker for poultry nutrition studies

We thank you for the opportunity to return a revised manuscript which we consider to be much improved by address to the criticisms raised in the review process. We thank our reviewers.

1.  Please ensure that your manuscript meets PLOS ONE's style requirements, including those for file naming. The PLOS ONE style templates can be found at

[https://journals.plos.org/plosone/s/file?id=wjVg/PLOSOne_formatting_sample_main_body.pdf](https://eur01.safelinks.protection.outlook.com/?url=https%3A%2F%2Fjournals.plos.org%2Fplosone%2Fs%2Ffile%3Fid%3DwjVg%2FPLOSOne_formatting_sample_main_body.pdf&data=05%7C01%7Cc.brearley%40uea.ac.uk%7C3a74460ca89b4189a39008dad66a22d3%7Cc65f8795ba3d43518a070865e5d8f090%7C0%7C0%7C638058049051015082%7CUnknown%7CTWFpbGZsb3d8eyJWIjoiMC4wLjAwMDAiLCJQIjoiV2luMzIiLCJBTiI6Ik1haWwiLCJXVCI6Mn0%3D%7C3000%7C%7C%7C&sdata=zB5AoVAaLzMDl4yg1ZWqKu%2Bj1%2Bt35crXhgkKZwTDqrM%3D&reserved=0)

and

[https://journals.plos.org/plosone/s/file?id=ba62/PLOSOne_formatting_sample_title_authors_affiliations.pdf](https://eur01.safelinks.protection.outlook.com/?url=https%3A%2F%2Fjournals.plos.org%2Fplosone%2Fs%2Ffile%3Fid%3Dba62%2FPLOSOne_formatting_sample_title_authors_affiliations.pdf&data=05%7C01%7Cc.brearley%40uea.ac.uk%7C3a74460ca89b4189a39008dad66a22d3%7Cc65f8795ba3d43518a070865e5d8f090%7C0%7C0%7C638058049051015082%7CUnknown%7CTWFpbGZsb3d8eyJWIjoiMC4wLjAwMDAiLCJQIjoiV2luMzIiLCJBTiI6Ik1haWwiLCJXVCI6Mn0%3D%7C3000%7C%7C%7C&sdata=%2Fb19QXdxMzS%2Bmpae5Pn%2FpO03QI0KgdC1DT7i9S1sCIE%3D&reserved=0)

Confirmed

2. Thank you for stating the following in the Competing Interests section:

Our Competing Interests section now reads, “The feeding trials described in this study were commissioned at Nottingham Trent University by AB Vista.  AB Vista had no role in conduct of the trial, data collection and analysis, decision to publish or preparation of the manuscript. This does not alter our adherence to PLOS ONE policies on sharing data and materials”

Additional Editor Comments:

I believe the experiment is carefully design, however, I and one of the reviewer is confused about your experimental design and analysis. For instance, readers don’t need to go to the accompanying paper to understand the experiment’s setup, therefore, should be explained in text. I don’t see a 2x4 in this description. It doesn’t mean it wasn’t a 2x4 arrangement, but it needs to be fully explained. What are the factors? If, for instance, we learn that the treatment arrangement was a 2x4. It begs the question of why the data in the tables were not analyzed as a 2x4. We hope you will consider our request in the revised manuscript.

We have altered the description of the trial design L26-30, L218-234 to make clear that the trial was designed to study the effect of titanium dioxide (TiO_2_) inclusion in diets with/without phytase. The tables of results and associated description L145-178 now include ANOVA to show effect of TiO_2_ (Titanium), diet and their interaction.

5. Review Comments to the Author

Please use the space provided to explain your answers to the questions above. You may also include additional comments for the author, including concerns about dual publication, research ethics, or publication ethics. (Please upload your review as an attachment if it exceeds 20,000 characters)

Reviewer #1: The work is interesting but there are too many issues with the premise and presentation of the work. Specific comments are shown below.
L16: Facile? I suggest the authors use another word. That makes the whole exercise superficial and meaningless.

Facile is replaced with ‘simple’

L16: total tract digestibility is a wrong word choice for several reasons. 1: when it is the total tract for poultry, it is not digestibility but retention. 2: index method is used not only for total tract…I suggest the authors simply state that it is used for digestibility and retention…

We stand corrected and have followed the suggestion with the sentence reading, ‘..of digestive tract retention and relative digestibility ..’.

L19: what is $b

Replaced with, ‘.. a billion dollar sector..’.

L19-26: this section is anticlimactic. Is it really necessary? The authors may consider rewording it, so it is less disappointing in the end.

We have retained the overall form of the Abstract to state our two main conclusions: those of new L24-26 and L31-34. We have, however, removed the ‘flowery’ sentence of the original L24-25, ‘ Consequently, the guiding tenets ..”.

L40-44: this premise may need to be adjusted. The index method is less laborious than total tract retention. Still, the use of markers also enables the determination of digestibility in the intestine, along intestinal sections, etc. lastly, the issue with poultry is not the contribution of microbial AA in the hindgut. This is applicable in other non-ruminants as well.

The reviewer is correct, we have modified the sentences accordingly.

L49-52: I think this is more suitable for the discussion section, and also must be substantiated with appropriate refs

We have left this aspect of the Introduction as written, because it sets the scene, but we have added extra references [new 23, 24, to pre-existing 2,3,4 and 22] and have provided a slightly more expansive discussion to the Discussion L183-199.

L87-90: this reads like a conclusion of the study. This should be deleted and moved to its appropriate place in the manuscript.

The text of L87-90 has been replaced with sentences new L98-104 that do not pre-empt the conclusions of the study.

L93: Target Feeds is not the manufacturer of TiO2; the authors should cite the manufacturer

We have scoured the poultry literature for statements of source of TiO_2_ used in feeding trials and can barely find any references to the source. Our source was Target Feeds, who have discontinued this product and no longer list it. We can offer no more information.

Fig 1: why are the InsP shown in panel B but not the others?

The labels have been added to all panels

Tables 1 and 2: tables are too busy and very cumbersome to read. I suggest 1. That the authors replace the within samples standard errors with pooled standard errors. 2. Instead of providing these high p values that were not significantly different, write “NS,” defined below the table as not significant.

The tables have been simplified. They have been modified to provide ANOVA for the eight diets. We have done so to address the principal criticisms of the manuscript – the explicit description of trial design. The description of the statistical analysis has been modified accordingly, new L272-276.

L178-81: I struggled hard to see how this fits in with the subject that this manuscript is trying to address. I suggest that the authors delete this because it detracts from their work.

We can see where the reviewer with a poultry science perspective is coming from. While much poultry research is very close to application, an awful lot of cell biological work on inositol phosphates is rather more distant from its translation. This paper brings the two together, we expect this to be read by animal nutritionists and cell biologists alike. For this reason and specifically because of the open science mission of PLOS ONE, we prefer to retain it.

L187-8: the point being made by the author is clear to me, but I don’t think that what the author claimed they found is what they found (or even investigated). The authors studied the recovery of InsP in diets with different Ti sources and found that TiO2 did not influence the InsP recovery. That is good to hear and shows that the old wisdom is not outdated (for now). However, the authors did not study (at least I didn’t see it) the effect of phytase, as in whether TiO2 affected the impact of phytase on InsP recovery. I see treatments with phytase (with or without Ti) – in this case, it seems that the subject is Ti, not phytase (in as much as both treatments have phytase). To show the interaction of phytase and Ti, there should at least be one more treatment (with Ti but without phytase). Or perhaps, the authors need to do a better job of explaining what they observed.

We take the reviewer’s point that our description of the trial design could have been better and that we did not draw out of our analysis a description of the effect of phytase, in part because it has been described many times before. We have done so now: the modified Tables 1 and 2 retain all the original measured values (mean and SEM) but now include the ANOVA to reveal lack of interaction of titanium dioxide (Titanium) and diet (phytase), lack of effect of Titanium and effect of diet (phytase). The description of the statistical analysis has been modified accordingly, new L272-276.

L190-5: I don’t see much in this conclusion. This reads more like a policy paper than the conclusion of rigorous scientific work. I like the work done here, but I don’t think the writing has done justice to the job done.

We have modified the closing sentences of the discussion, new L193-197 to put our work in context of other phytase studies (ref 10, 11, 13-15). We have reduced the ‘policy’ aspect of the discussion and finish up, new L197-204, with a statement encouraging others to use TiO_2_ both as marker and as an aid to extraction of inositol phosphates from tissues of poultry. We cite [8], the first study to use TiO_2_ for the latter purpose.

L213-5: I appeal to the authors to help me here. I don’t need to go to the accompanying paper to understand the experiment’s setup. I don’t see a 2x4 in this description. It doesn’t mean it wasn’t a 2x4 arrangement, but it needs to be fully explained. What are the factors?

We hope we have provided greater clarity in our description of the design of the trial and in the presentation of the results with associated ANOVA Tables 1 and 2, which specifically identifies the factors, Titanium and diet. The description of the statistical analysis has been modified accordingly, new L272-276.

L213-5: now that we learn that the treatment arrangement was a 2x4. It begs the question of why the data in the tables were not analyzed as a 2x4.

We hope we have provided greater clarity in our description of the design of the trial and in the presentation of the results with associated ANOVA Tables 1and 2, which specifically identifies the factors, Titanium and diet. The description of the statistical analysis has been modified accordingly, new L272-276.

L232: finely ground is not defined. Be more specific, please.

We have added detail of the use of a coffee grinder, albeit without particle size analysis, and have provided further detail of the freeze-drying with reference [25] to the AOAC method, new L242-245.

L261-7: I didn’t see any evidence of Tukey separation in the tables presented here. But a more significant problem is using the T test to separate the means. Isn’t that what Tukey is supposed to do? Did the authors consider that the T-test is not used when multiple treatments are involved because there is a loss in statistical power with each sequence of comparisons? The correct thing to do would be to have analyzed the data as a 2x4 (in as much as that was the treatment arrangement). Unless, of course, I missed something.

The reviewer is correct, we have provided the ANOVA for clarity. The description of the statistical analysis has been modified accordingly, new L272-276.

Reviewer #2: This is a nice study which it worth to be known by the animal nutritionist that usually trust on digestibility values estimated by the index marker ratio, using TiO2 as one of the most used markers. The hypothesis is clear and well justified, and the experimental protocol allows to identify, if they would have existed, likely interference or interactions on the phytase activity in digesta.

We thank the reviewer.

There are only one minor comment that probably it has a proper explanation
Line 43.- Please, explain or delete the following statement “particularly in poultry where the contribution of microbial amino acids also confounds direct balance studies” Which is it the microbial interference that affects to direct studies and not to the marker index ratio estimate.

We have removed the statement.

Finally, we thank our reviewers and editor for their consideration of our manuscript.

For the purpose of open access, the author has applied a Creative Commons Attribution (CC BY) to any Author Accepted Manuscript version arising.

Yours sincerely

Charles Brearley
