# Supplementary material for: Accentuating the positive and eliminating the negative: Efficacy of TiO2 as digestibility index marker for poultry nutrition studies
Source: PLoS One. 2023 Jun 26;18(6):e0284724. doi: 10.1371/journal.pone.0284724 (PMC10292697; doi:10.1371/journal.pone.0284724)
Supplement: Attachment — Submitted filename: Response to reviewers R2.docx [file pone.0284724.s005.docx]

20 March 2023

Dear Aziz ur Rahman Muhammad,

Please consider our revised manuscript for publication in PLOS ONE. Our detailed response to the comments arising from review of our previous revision (R1) are given below where specific revisions have been requested.

Dear Dr. Brearley,

Thank you for submitting your manuscript to PLOS ONE. After careful consideration, we feel that it has merit but does not fully meet PLOS ONE’s publication criteria as it currently stands. Therefore, we invite you to submit a revised version of the manuscript that addresses the points raised during the review process.
Dear Authors, 
I still believe that manuscript require to improve the statistical part as suggested by reviewer. after improving statistical part, manuscript will be ready for publication. I invite authors to address the issues of reviewer please. 
Please submit your revised manuscript by Mar 24 2023 11:59PM. If you will need more time than this to complete your revisions, please reply to this message or contact the journal office at [plosone@plos.org](mailto:plosone@plos.org). When you're ready to submit your revision, log on to [https://www.editorialmanager.com/pone/](https://eur01.safelinks.protection.outlook.com/?url=https%3A%2F%2Fwww.editorialmanager.com%2Fpone%2F&data=05%7C01%7CC.Sprigg%40uea.ac.uk%7C9d9ce24f6953491cd20408db199e11ce%7Cc65f8795ba3d43518a070865e5d8f090%7C0%7C0%7C638131938904845785%7CUnknown%7CTWFpbGZsb3d8eyJWIjoiMC4wLjAwMDAiLCJQIjoiV2luMzIiLCJBTiI6Ik1haWwiLCJXVCI6Mn0%3D%7C3000%7C%7C%7C&sdata=TZk11%2BYmxBMOAm0rr4Q0bzsRxnrna%2Bk8ZULZ6JYulmY%3D&reserved=0) and select the 'Submissions Needing Revision' folder to locate your manuscript file.

Please include the following items when submitting your revised manuscript:

- A rebuttal letter that responds to each point raised by the academic editor and reviewer(s). You should upload this letter as a separate file labeled 'Response to Reviewers'.
- A marked-up copy of your manuscript that highlights changes made to the original version. You should upload this as a separate file labeled 'Revised Manuscript with Track Changes'.
- An unmarked version of your revised paper without tracked changes. You should upload this as a separate file labeled 'Manuscript'.

If you would like to make changes to your financial disclosure, please include your updated statement in your cover letter. Guidelines for resubmitting your figure files are available below the reviewer comments at the end of this letter.

If applicable, we recommend that you deposit your laboratory protocols in protocols.io to enhance the reproducibility of your results. Protocols.io assigns your protocol its own identifier (DOI) so that it can be cited independently in the future. For instructions see: [https://journals.plos.org/plosone/s/submission-guidelines#loc-laboratory-protocols](https://eur01.safelinks.protection.outlook.com/?url=https%3A%2F%2Fjournals.plos.org%2Fplosone%2Fs%2Fsubmission-guidelines%23loc-laboratory-protocols&data=05%7C01%7CC.Sprigg%40uea.ac.uk%7C9d9ce24f6953491cd20408db199e11ce%7Cc65f8795ba3d43518a070865e5d8f090%7C0%7C0%7C638131938904845785%7CUnknown%7CTWFpbGZsb3d8eyJWIjoiMC4wLjAwMDAiLCJQIjoiV2luMzIiLCJBTiI6Ik1haWwiLCJXVCI6Mn0%3D%7C3000%7C%7C%7C&sdata=YTJbhzagKlqsD%2B%2BFIZGJr4UZpEpsktWHaUOgCQ1Hp%2FI%3D&reserved=0). Additionally, PLOS ONE offers an option for publishing peer-reviewed Lab Protocol articles, which describe protocols hosted on protocols.io. Read more information on sharing protocols at [https://plos.org/protocols?utm_medium=editorial-email&utm_source=authorletters&utm_campaign=protocols](https://eur01.safelinks.protection.outlook.com/?url=https%3A%2F%2Fplos.org%2Fprotocols%3Futm_medium%3Deditorial-email%26utm_source%3Dauthorletters%26utm_campaign%3Dprotocols&data=05%7C01%7CC.Sprigg%40uea.ac.uk%7C9d9ce24f6953491cd20408db199e11ce%7Cc65f8795ba3d43518a070865e5d8f090%7C0%7C0%7C638131938904845785%7CUnknown%7CTWFpbGZsb3d8eyJWIjoiMC4wLjAwMDAiLCJQIjoiV2luMzIiLCJBTiI6Ik1haWwiLCJXVCI6Mn0%3D%7C3000%7C%7C%7C&sdata=mnaov4mEsi7N0hC9lwyH2STDkRATLOti92cBPowCelE%3D&reserved=0).

We look forward to receiving your revised manuscript.

Kind regards,

Aziz ur Rahman Muhammad
Academic Editor
PLOS ONE

Additional Editor Comments (if provided):

Dear Authors,

I still believe that authors are required to improve the statistical part as suggested by reviewer. after improving statistical part, manuscript will be ready for publication. I invite authors to address the issues of reviewer please.


[Note: HTML markup is below. Please do not edit.]

Reviewers' comments:

Reviewer's Responses to Questions

**Comments to the Author**

1. If the authors have adequately addressed your comments raised in a previous round of review and you feel that this manuscript is now acceptable for publication, you may indicate that here to bypass the “Comments to the Author” section, enter your conflict of interest statement in the “Confidential to Editor” section, and submit your "Accept" recommendation.

Reviewer #1: (No Response)

2. Is the manuscript technically sound, and do the data support the conclusions?

The manuscript must describe a technically sound piece of scientific research with data that supports the conclusions. Experiments must have been conducted rigorously, with appropriate controls, replication, and sample sizes. The conclusions must be drawn appropriately based on the data presented.

Reviewer #1: Partly

3. Has the statistical analysis been performed appropriately and rigorously?

Reviewer #1: No

4. Have the authors made all data underlying the findings in their manuscript fully available?

The [PLOS Data policy](https://eur01.safelinks.protection.outlook.com/?url=http%3A%2F%2Fwww.plosone.org%2Fstatic%2Fpolicies.action%23sharing&data=05%7C01%7CC.Sprigg%40uea.ac.uk%7C9d9ce24f6953491cd20408db199e11ce%7Cc65f8795ba3d43518a070865e5d8f090%7C0%7C0%7C638131938904845785%7CUnknown%7CTWFpbGZsb3d8eyJWIjoiMC4wLjAwMDAiLCJQIjoiV2luMzIiLCJBTiI6Ik1haWwiLCJXVCI6Mn0%3D%7C3000%7C%7C%7C&sdata=fAHh9nU%2FrlfGocb7YL4nRGPCSqqRlb6jIeb2x5QKAhc%3D&reserved=0) requires authors to make all data underlying the findings described in their manuscript fully available without restriction, with rare exception (please refer to the Data Availability Statement in the manuscript PDF file). The data should be provided as part of the manuscript or its supporting information, or deposited to a public repository. For example, in addition to summary statistics, the data points behind means, medians and variance measures should be available. If there are restrictions on publicly sharing data—e.g. participant privacy or use of data from a third party—those must be specified.

Reviewer #1: Yes

5. Is the manuscript presented in an intelligible fashion and written in standard English?

PLOS ONE does not copyedit accepted manuscripts, so the language in submitted articles must be clear, correct, and unambiguous. Any typographical or grammatical errors should be corrected at revision, so please note any specific errors here.

Reviewer #1: No

6. Review Comments to the Author

Please use the space provided to explain your answers to the questions above. You may also include additional comments for the author, including concerns about dual publication, research ethics, or publication ethics. (Please upload your review as an attachment if it exceeds 20,000 characters)

Reviewer #1: 1. I still struggle with the stat analysis done in this manuscript. The ANOVA output presented in the tables are totally unhelpful. There are only one set of ANOVA whereas there are 6 sets of dependent variables. So the question is what set of the dependent variables are the one ANOVA for. I must say that the readers don't need the ANOVA output but the p values. I suggest speaking with a statistician to explore the best way to do the stat analysis.

2. Still on the analysis, although as pointed out earlier, only set of p values are provided, and it is unclear what those p values are for - i hope that this is addressed. But looking at those p values provided, I notice that only the main effect of the factor "diet" is significant. When this is the case, the authors should rearrange the data in the table to show the main effects means. Namely provide the means for the two levels of phytase and the two levels of Ti (i.e. with or without Ti). Of course, if there are any interaction of factors for any of the dependent variables, the simple (all 8 means) should be listed and the means separated as appropriate with Tukey or similar.

Dear Aziz ur Rahman Muhammad,

Please consider our revised manuscript for publication in PLOS One. The manuscript now bears the additional author, Philip T. Leftwich, for his contribution to the statistical analysis of data described in the manuscript.

To clear up any further confusion, previous reference to 2 x 4 factorial trial design has been replaced with simple reference to there being 8 treatments. For ease of understanding for the reader, a table has now been included in the Materials and Methods further describing each diet/treatment (Table 1).

We have reported the variance explained by the interaction terms, and as this was a non-significant result, have dropped this from the final model in favour of an additive two-way ANOVA. In order to simplify the presentation of results, we have reported ANOVA results in text (with the necessary F values and degrees of freedom required to accurately interpret p-values) and have replaced the sum of squares table with a presentation of means and standard errors for all treatment groups. As Ti was not a significant effect, we elected to show mean summaries for all 8 treatment groups.

Additionally, tables have been simplified. They now show the means and SEM for all treatments. Means were tested by Two-Way ANOVA – as interaction was found to be not significant, means were tested again with interaction removed from the model to generate the p values presented. Follow up Tukey tests on diets were not reported in this manuscript as the effect of phytase and inositol supplementation compared to the Control for these data sets has been reported previously in Sprigg *et al.* (2022), and the effect of phytase was not the focus of this report. Consequently, ANOVA values have been removed from the results tables (Tables 2 & 3) and relevant information has been included in the narrative (Results section).

3. On the matter of TiO2 source, is it really necessary to indicate the source? TiO_2_ is sold by chemical suppliers like Sigma Aldrich, Fisher etc, not the feed manufacturer. This author felt it appropriate to include the supplier of the TiO_2_ as it differentiates from titanium manufactured and sold as a fixed particle size specifically for use in purifying phosphopeptides in cell biology research (Titansphere) from mixed particle size TiO_2_ supplied for use as a digestibility index marker (purchased through Target Feeds with no listed manufacturer).

Yours sincerely

Charles Brearley

7. PLOS authors have the option to publish the peer review history of their article ([what does this mean?](https://eur01.safelinks.protection.outlook.com/?url=https%3A%2F%2Fjournals.plos.org%2Fplosone%2Fs%2Feditorial-and-peer-review-process%23loc-peer-review-history&data=05%7C01%7CC.Sprigg%40uea.ac.uk%7C9d9ce24f6953491cd20408db199e11ce%7Cc65f8795ba3d43518a070865e5d8f090%7C0%7C0%7C638131938904845785%7CUnknown%7CTWFpbGZsb3d8eyJWIjoiMC4wLjAwMDAiLCJQIjoiV2luMzIiLCJBTiI6Ik1haWwiLCJXVCI6Mn0%3D%7C3000%7C%7C%7C&sdata=mhBerimpYqYNGJLP618xK8dmfqtRbEZMrjEm%2FG7jYEo%3D&reserved=0)). If published, this will include your full peer review and any attached files.


If you choose “no”, your identity will remain anonymous but your review may still be made public.


**Do you want your identity to be public for this peer review?** For information about this choice, including consent withdrawal, please see our [Privacy Policy](https://eur01.safelinks.protection.outlook.com/?url=https%3A%2F%2Fwww.plos.org%2Fprivacy-policy&data=05%7C01%7CC.Sprigg%40uea.ac.uk%7C9d9ce24f6953491cd20408db199e11ce%7Cc65f8795ba3d43518a070865e5d8f090%7C0%7C0%7C638131938904845785%7CUnknown%7CTWFpbGZsb3d8eyJWIjoiMC4wLjAwMDAiLCJQIjoiV2luMzIiLCJBTiI6Ik1haWwiLCJXVCI6Mn0%3D%7C3000%7C%7C%7C&sdata=XsqSP0Yr29utGA%2FOUtp0GVhQyD%2FgkQYe4NZr%2Bn1u9bU%3D&reserved=0).

Reviewer #1: No
